# Associations of Serum CIRP and CXCL5 With Prognosis and Neutrophil Extracellular Traps in Pediatric *Mycoplasma pneumoniae* Pneumonia

**DOI:** 10.1111/crj.70231

**Published:** 2026-09-24

**Authors:** Litao Wan, Bolin Liu, Ning Xie, Jianhua Li, Ting Su, Liyan Zhang, Xiaohua Wang, Shiding Gao

**Affiliations:** ^1^ Department of Infectious Diseases The 908th Hospital of Chinese People's Liberation Army Joint Logistic Support Force Nanchang Jiangxi China; ^2^ Department of Otorhinolaryngology Head and Neck Surgery The 908th Hospital of Chinese People's Liberation Army Joint Logistic Support Force Nanchang Jiangxi China; ^3^ Department of Health and Epidemic Prevention Jiangxi General Hospital of the Chinese People's Armed Police Force Nanchang Jiangxi China; ^4^ Department of General Surgery The 908th Hospital of Chinese People's Liberation Army Joint Logistic Support Force Nanchang Jiangxi China

**Keywords:** cold‐inducible RNA‐binding protein, C‐X‐C motif chemokine ligand 5, *Mycoplasma pneumoniae* pneumonia, neutrophil extracellular traps, prognosis

## Abstract

**Objective:**

This study aimed to investigate the associations of serum cold‐inducible RNA‐binding protein (CIRP) and C‐X‐C motif chemokine ligand 5 (CXCL5) with prognosis in children with *Mycoplasma pneumoniae* pneumonia (MPP) and their relationship with neutrophil extracellular traps (NETs).

**Methods:**

A total of 485 children with MPP admitted between January 2022 and March 2025 were enrolled. Patients were categorized into good‐prognosis (*n* = 379) and poor‐prognosis (*n* = 106) groups based on therapeutic efficacy at 1 week after treatment completion. Serum levels of CIRP, CXCL5, and NET markers (myeloperoxidase‐DNA complex [MPO‐DNA] and citrullinated histone H3 [CitH3]) were measured. Multivariable logistic regression was used to analyze factors influencing prognosis. Receiver operating characteristic (ROC) curve analysis was performed to evaluate predictive value. Spearman correlation analysis was used to examine the correlations of CIRP and CXCL5 with NET markers.

**Results:**

Compared with the good‐prognosis group, the poor‐prognosis group exhibited longer fever duration, higher neutrophil percentage, elevated levels of D‐dimer, C‐reactive protein (CRP), and interleukin‐6 (IL‐6), higher rates of multilobar involvement and pleural effusion, and more frequent use of systemic glucocorticoids, intravenous immunoglobulin (IVIG), fiberoptic bronchoscopy with bronchoalveolar lavage, and second‐line antimicrobial agents (all *p* < 0.05). Serum CIRP and CXCL5 levels were significantly higher in the poor‐prognosis group (both *p* < 0.001). Multivariable logistic regression analysis demonstrated that neutrophil percentage, MP antibody titer, D‐dimer, CRP, IL‐6, extent of pulmonary involvement, pleural effusion, CIRP, and CXCL5 were independent risk factors for poor prognosis (all *p* < 0.05). ROC analysis showed areas under the curve (AUCs) of 0.733 for CIRP, 0.752 for CXCL5, and 0.796 for their combination in predicting poor prognosis, with the combination showing superior predictive performance. Levels of NET markers were significantly elevated in the poor‐prognosis group (both *p* < 0.001), and both CIRP and CXCL5 were positively correlated with MPO‐DNA and CitH3.

**Conclusions:**

Elevated serum CIRP and CXCL5 levels are associated with poor prognosis in children with MPP and correlate with NET markers. Combined detection may facilitate early identification of high‐risk patients; however, whether these biomarkers influence disease progression through modulation of NET requires further investigation.

## Graphical abstract

This study investigated serum levels of cold‐inducible RNA‐binding protein (CIRP) and C‐X‐C motif chemokine ligand 5 (CXCL5) in 485 children with *Mycoplasma pneumoniae* pneumonia. Elevated levels of both biomarkers were independently associated with poor prognosis and positively correlated with neutrophil extracellular trap markers, suggesting their prognostic utility.

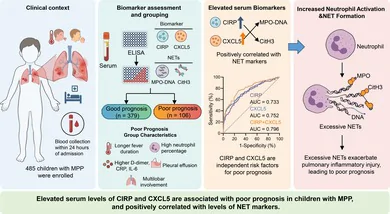

## 1 Introduction

*M. pneumoniae* pneumonia (MPP) is a common acute respiratory tract infection in children. Although most pediatric patients have a good prognosis, some may progress to severe pneumonia complicated by multiple organ dysfunction, posing a serious threat to their survival and well‐being [1, 2, 3, 4]. Therefore, early identification of children at high risk for poor prognosis and further elucidation of the pathogenesis are of great significance for guiding individualized clinical intervention.

Neutrophil extracellular traps (NETs) are weblike structures released by neutrophils and composed of DNA fibers, histones, and granule proteins. They play a critical role in capturing and killing pathogens; however, excessive NET formation can contribute to the development and progression of various diseases through mechanisms including direct cytotoxicity and activation of pro‐inflammatory signaling [5, 6]. Recent studies have demonstrated that NETs play an important role in the pathogenesis of MPP. M. pneumoniae infection can induce excessive NET release from neutrophils, exacerbating pulmonary inflammatory injury. NET levels are closely associated with disease severity and clinical outcomes in MPP. Targeted regulation of NET formation and degradation may offer a novel therapeutic strategy for improving prognosis in MPP [7, 8, 9].

Cold‐inducible RNA‐binding protein (CIRP) is a stress‐inducible RNA‐binding protein whose expression is upregulated in various pathological conditions, including inflammation, infection, and oxidative stress [10]. In animal models of bacterial pneumonia, CIRP expression levels increase with bacterial load and correlate positively with both the severity of lung tissue inflammation and mortality [11]. In sepsis models, CIRP has been shown to directly induce the formation of NETs by upregulating peptidylarginine deiminase 4 (PAD4) expression, thereby causing acute lung injury; blockade of CIRP or inhibition of PAD4 significantly reduces NET formation and improves outcomes [12]. However, CIRP expression in MPP and its relationship with NETs and prognosis remain unexplored.

C‐X‐C motif chemokine ligand 5 (CXCL5) is a CXC chemokine primarily secreted by epithelial cells, endothelial cells, and macrophages. Through specific binding to its receptor CXCR2, CXCL5 potently chemoattracts neutrophils to inflammatory sites, exacerbating inflammatory responses [13, 14]. Studies have shown that CXCL5 expression can directly disrupt alveolar‐epithelial barrier permeability, aggravating acute lung injury [15], whereas genetic ablation of CXCL5 significantly attenuates pulmonary inflammation [16]. However, altered expression of CXCL5 in MPP and its relationship with NET formation and disease prognosis remain unclear.

Therefore, this study aims to measure serum levels of CIRP, CXCL5, and NET‐related markers in children with MPP, analyze their correlation with prognosis, and investigate the association between CIRP, CXCL5, and NETs, thereby providing a novel theoretical basis and biomarkers for early identification of high‐risk children and the development of targeted intervention strategies.

## 2 Methods

### 2.1 Study Participants

This study was designed as a single‐center prospective cohort study. The sample size was estimated based on a previous study [17], which reported that the incidence of poor prognosis in children with MPP was 20.23%. With a two‐sided alpha of 0.05 (1 − *α* = 0.95) and an absolute precision of 5%, the minimum required sample size was calculated to be 268 using PASS 15.0 software. Accounting for an anticipated 10% loss to follow‐up, the target sample size was determined to be 295. During the study period, to enhance the stability of multivariable logistic regression analysis and ensure the reliability of subgroup analyses, a total of 485 children with MPP were ultimately enrolled. Based on prognosis, they were categorized into a good‐prognosis group (*n* = 379) and a poor‐prognosis group (*n* = 106).

Inclusion criteria: (1) Diagnosis of MPP according to established diagnostic criteria [18], requiring fulfillment of all of the following: (a) Clinical manifestations: fever, irritative dry cough, the absence of obvious pulmonary signs in the early stage, and adventitious sounds or signs of consolidation upon progression; (b) Imaging findings: chest radiography or computed tomography showing increased bronchovascular markings, patchy infiltrates, ground‐glass opacities, or consolidation; and (c) etiological confirmation (any of the following): positive *M. pneumoniae* (MP)‐DNA or MP‐RNA in respiratory specimens (throat swab, sputum, or bronchoalveolar lavage fluid); a single serum MP‐IgM titer of ≥ 1:160 by particle agglutination (PA) assay; a ≥ 4‐fold increase in serum MP‐IgG titer in paired sera, or positive MP culture. (2) Age 3–12 years; (3) first‐time diagnosis; (4) complete clinical data available; and (5) written informed consent obtained from the child's legal guardian. Exclusion criteria: (1) Co‐infection with other acute or chronic pathogens; (2) etiologically confirmed infection with other acute or chronic bacterial, viral, or fungal pathogens (e.g., influenza virus, adenovirus, and *Streptococcus pneumoniae* ); (3) underlying pulmonary conditions including bronchial asthma, pulmonary tuberculosis, bronchiectasis, congenital heart disease, or pulmonary fibrosis; (4) severe cardiac, hepatic, or renal dysfunction; (5) autoimmune diseases, primary immunodeficiency disorders, or allergic diseases; (6) hematological disorders or malignancies; (7) prior antibiotic therapy or other medications for pneumonia before enrollment; (8) psychiatric or cognitive disorders precluding cooperation with treatment and examinations; and (9) incomplete clinical data.

### 2.2 Treatment

All children with MPP received routine symptomatic and supportive care upon admission, including antipyretic therapy, antitussive and expectorant therapy, and oxygen therapy. Additionally, all patients were uniformly treated with intravenous azithromycin at 10 mg·kg^−1^·day^−1^ for 1 week. For those with disease progression or inadequate response to initial therapy, the attending physician determined whether to add the following adjunctive therapies according to the evidence‐based guideline for the diagnosis and treatment of *M. pneumoniae* pneumonia in children (2023) [18]: (1) systemic glucocorticoids (methylprednisolone at an initial dose of 1–2 mg·kg^−1^·day^−1^, escalated to 4–6 mg·kg^−1^·day^−1^ in severe cases, with gradual tapering after defervescence); (2) intravenous immunoglobulin (IVIG) at 1 g·kg^−1^·day^−1^ for 1–2 days, primarily for severe extrapulmonary complications; (3) fiberoptic bronchoscopy with bronchoalveolar lavage, indicated for suspected plastic bronchitis, mucus plug obstruction, or extensive lung consolidation with failure of resolution; and (4) second‐line antimicrobial agents (doxycycline or minocycline for children aged ≥ 8 years; fluoroquinolones for severely ill children, with informed consent obtained from guardians).

### 2.3 Clinical Data Collection

Demographic and clinical characteristics, including sex, age, body mass index (BMI), body temperature at admission, duration of fever, and length of hospital stay, were retrieved from the hospital electronic medical record system. Concurrently, chest radiography was performed, with the extent of pulmonary involvement and the presence of pleural effusion independently assessed by two senior radiologists.

### 2.4 Laboratory Examinations

Fasting venous blood samples (5 mL) were collected within 24 h of admission, left at room temperature for 30 min, and centrifuged at 1000 ×*g* for 10 min to separate serum. Serum samples were stored at −80°C until analysis, with no more than two freeze–thaw cycles. The following assays were performed:

1. Complete blood count parameters, including platelet count, neutrophil percentage, white blood cell count, and hemoglobin, were measured using an automated hematology analyzer (bc‐6000; Mindray, China). MP antibody titers were determined using a commercial kit (particle agglutination assay; Zhuhai Livzon, China). D‐dimer and C‐reactive protein (CRP) levels were measured using an automated biochemistry analyzer (BS‐2800M; Mindray, China).
2. Lactate dehydrogenase (LDH), interleukin‐6 (IL‐6), CIRP, CXCL5, and NET‐related markers, including myeloperoxidase‐DNA (MPO‐DNA) complex and citrullinated histone H3 (CitH3), were measured by enzyme‐linked immunosorbent assay (ELISA). The following ELISA kits were used: LDH (catalog no. ml106625), IL‐6 (catalog no. ml058097), CIRP (catalog no. ml105498), CXCL5 (catalog no. ml058425), MPO‐DNA (catalog no. ml342558), and CitH3 (catalog no. ml106856), all purchased from Shanghai Enzyme‐linked Biotechnology Co. Ltd.

### 2.5 ELISA Quality Control and Blinding Design

Serum samples and all reagents were equilibrated to room temperature before analysis. ELISA was performed strictly per the manufacturer's instructions: Standards and pretreated serum samples were added to antibody‐coated plates, followed by incubation, washing, addition of biotinylated detection antibody or HRP‐labeled antibody, further incubation and washing, addition of TMB chromogenic substrate, termination with stop solution, and absorbance measurement at 450 nm. Concentrations were calculated from four‐parameter logistic standard curves. Each sample was analyzed in duplicate, and mean values were used for analysis. Both intraassay and interassay coefficients of variation (CV) were < 10%.

A blinded design was employed throughout the study: Samples were randomly coded by personnel not involved in the study, and both technicians and data analysts were blinded to group allocation.

### 2.6 Prognostic Assessment and Grouping

All children with MPP underwent outpatient follow‐up 1 week after treatment completion, and treatment response was evaluated according to the reference [19]. Patients who were afebrile for ≥ 72 h (three consecutive days), had significant improvement or resolution of cough and sputum, and had chest CT demonstrating ≥ 30% resolution of pulmonary consolidation, or no new lesions were assigned to the good‐prognosis group (*n* = 379). Patients with persistent or recurrent fever (> 39°C for ≥ 5 days or total fever duration > 7 days), unchanged or worsening cough and sputum, disease progression (development of atelectasis, necrotizing pneumonia, pleural effusion, etc.), or extrapulmonary complications (neurological, hematologic, or mucocutaneous involvement, etc.) were assigned to the poor‐prognosis group (*n* = 106).

### 2.7 Statistical Analysis

Statistical analyses were performed using SPSS 26.0 (IBM, Armonk, NY, USA), and graphs were created using GraphPad Prism version 9.5. Normality of continuous variables was examined using the Kolmogorov–Smirnov test. Normally distributed data were expressed as mean ± standard deviation, and between‐group comparisons were performed using the *t*‐test. Nonnormally distributed data were presented as median and interquartile range [*M* (P25, P75)], and between‐group comparisons were conducted using the Mann–Whitney *U* test. Categorical variables were expressed as numbers and percentages (%), and between‐group comparisons were performed using the *χ* ^2^ test. Multivariable logistic regression with a forward stepwise method (Forward: LR; entry criterion, *p* < 0.05; removal criterion, *p* > 0.10) was used to identify prognostic factors in children with MPP. Nonnormally distributed continuous variables were natural log‐transformed prior to inclusion in the analysis. The variance inflation factor (VIF) was used to assess multicollinearity, with a VIF > 5 indicating the presence of multicollinearity. Receiver operating characteristic (ROC) curve analysis was performed to evaluate the predictive performance of CIRP, CXCL5, and their combined model. The combined model was a logistic regression model incorporating both CIRP and CXCL5. ROC curves were plotted based on predicted probabilities. Optimal cutoff values were determined using the maximum Youden index, and sensitivity, specificity, and 95% CI were reported. Spearman correlation analysis was performed to determine the correlations of serum CIRP and CXCL5 with NET markers (MPO‐DNA and CitH3). A *p*‐value < 0.05 was considered statistically significant.

## 3 Results

### 3.1 Comparison of Clinical Characteristics Between the Good‐Prognosis Group and the Poor‐Prognosis Group

No statistically significant differences were observed between the two groups in terms of sex, age, BMI, body temperature at admission, or length of hospital stay (all *p* > 0.05). Relative to the good‐prognosis group, the poor‐prognosis group demonstrated prolonged duration of fever, elevated neutrophil percentage, and increased concentrations of D‐dimer, CRP, and IL‐6, as well as higher proportions of multilobar involvement and pleural effusion. The proportions of children receiving systemic glucocorticoids, IVIG, fiberoptic bronchoscopy with bronchoalveolar lavage, and second‐line antimicrobial agents were also significantly higher in the poor‐prognosis group (all *p* < 0.05) (Table 1).

**TABLE 1:** Comparison of clinical characteristics between the good‐prognosis group and the poor‐prognosis group.

Variable	Good‐prognosis group (*n* = 379)	Poor‐prognosis group (*n* = 106)	*t*/*Z*/*χ* ^2^	*p*
Sex, *n* (%)			0.203^c^	0.652
Male	206 (54.35)	55 (51.89)		
Female	173 (45.65)	51 (48.11)		
Age, years	7.00 (6.00, 8.00)	7.00 (6.00, 8.00)	−0.442^b^	0.658
BMI, kg/m^2^	16.50 ± 2.22	16.38 ± 1.92	0.483^a^	0.629
Body temperature at admission,°C	37.45 ± 0.86	37.61 ± 0.91	−1.704^a^	0.089
Duration of fever, days	5.00 (4.00, 5.00)	7.00 (6.00, 8.00)	−12.302^a^	< 0.001
Length of hospital stay, days	8.00 (7.00, 10.00)	8.00 (7.00, 11.00)	−0.650^a^	0.516
Platelet count, ×10⁹/L	203.56 ± 22.41	201.37 ± 26.58	0.853^a^	0.394
Neutrophil percentage, %	58.00 (51.00, 64.00)	72.75 (64.05, 80.65)	−10.123^a^	< 0.001
White blood cell count, ×10⁹/L	7.26 ± 2.23	7.42 ± 2.52	−0.633^a^	0.527
Hemoglobin, g/L	106.51 ± 15.21	103.91 ± 19.12	1.467^a^	0.143
MP antibody titer, *n* (%)			9.351^c^	0.002
< 1:160	329 (86.81)	86 (81.13)		
≥ 1:160	50 (13.19)	20 (18.86)		
D‐dimer, mg/L	1.43 (1.20, 1.72)	1.80 (1.50, 2.23)	−7.469^a^	< 0.001
LDH, ng/mL	79.42 ± 8.55	80.55 ± 9.11	−1.191^a^	0.234
CRP, mg/L	20.67 (17.87, 23.77)	39.14 (35.47, 43.99)	−15.581^a^	< 0.001
IL‐6, pg/mL	102.73 ± 44.37	138.80 ± 30.75	−7.853^a^	< 0.001
Extent of pulmonary involvement, *n* (%)			11.049^c^	0.004
Single lobe	83 (21.89)	15 (14.15)		
Two lobes	136 (35.88)	27 (25.47)		
More than two lobes	160 (42.22)	64 (60.38)		
Pleural effusion, *n* (%)			18.338^c^	< 0.001
Present	66 (17.41)	39 (36.79)		
Absent	313 (82.58)	67 (63.21)		
Adjuvant therapies, *n* (%)				
Systemic glucocorticoids	45 (11.87)	55 (51.90)	81.029^c^	< 0.001
IVIG	10 (2.64)	20 (18.87)	37.598^c^	< 0.001
Fiberoptic bronchoscopy with bronchoalveolar lavage	14 (3.69)	40 (37.74)	97.016^c^	< 0.001
Second‐line antimicrobial agents	5 (1.32)	32 (30.19)	97.968^c^	< 0.001

^a^ indicates *t*‐value.

^b^ indicates *Z*‐value.

^c^ indicates *χ* ^2^ value.

### 3.2 Comparison of Serum CIRP and CXCL5 Levels Between the Good‐Prognosis and the Poor‐Prognosis Groups

**FIGURE 1:**
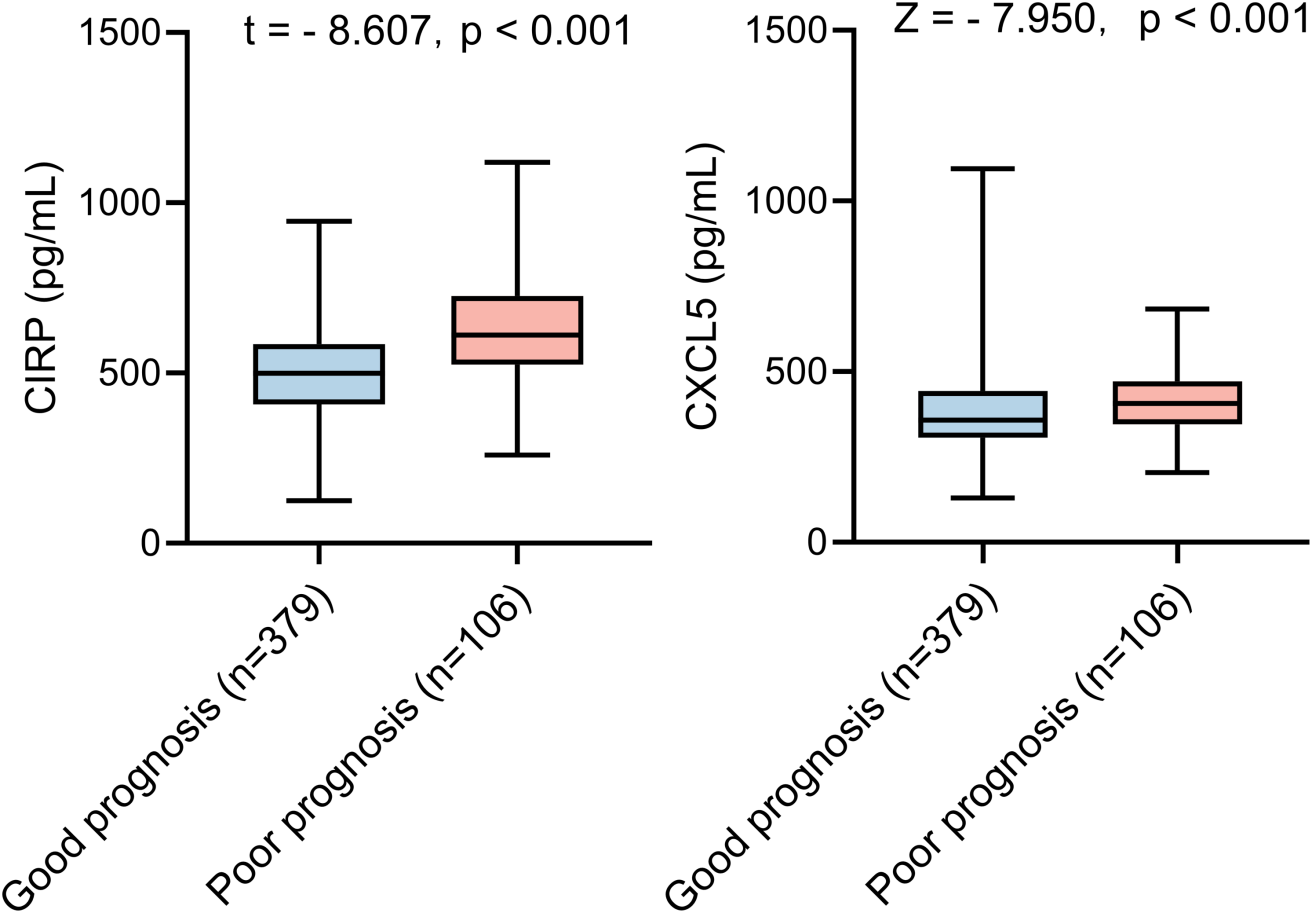
Comparison of serum CIRP and CXCL5 levels between the good‐prognosis group and the poor prognosis group.

Compared with the good‐prognosis group, children in the poor‐prognosis group showed significantly elevated serum CIRP and CXCL5 levels (both *p* < 0.001) (Figure 1).

### 3.3 Multivariable Logistic Regression for Poor Prognosis in Children With MPP

Prognosis was used as the dependent variable (*Poor prognosis*, 1; *Good prognosis*, 0). Pre‐treatment baseline variables that showed statistically significant differences in univariate analysis were entered into a multivariable logistic regression model. Adjuvant therapies (systemic glucocorticoids, IVIG, bronchoscopy, and second‐line antimicrobial agents) were dynamically adjusted during the course of treatment and exhibited bidirectional causal associations with prognosis, failing to meet the temporal precedence requirement for independent variables in predictive models; furthermore, fever duration overlapped with the outcome definition. To avoid incorporation bias, these variables were excluded from the final model. Multivariable logistic regression analysis revealed that neutrophil percentage, MP antibody titer, D‐dimer, CRP, IL‐6, extensive pulmonary involvement, the presence of pleural effusion, CIRP, and CXCL5 were all independent risk factors for poor prognosis in children with MPP. The VIF for each variable was < 2, indicating no significant multicollinearity (Table 2).

**TABLE 2:** Multivariable logistic regression for predictors of poor prognosis in children with MPP.

Variable	*β*	S.E	Wald	*p*	OR	95% CI	VIF
Neutrophil percentage^a^	0.116	0.013	82.334	< 0.001	1.123	1.095–1.152	1.219
MP antibody titer	0.840	0.276	9.245	0.002	2.317	1.348–3.982	1.028
D‐dimer^a^	0.847	0.157	28.917	< 0.001	2.332	1.713–3.175	1.187
CRP^a^	0.897	0.217	17.137	< 0.001	2.452	1.604–3.750	1.539
IL‐6	0.023	0.004	36.53	< 0.001	1.023	1.016–1.031	1.122
Extent of pulmonary involvement	0.482	0.159	9.254	0.002	1.620	1.187–2.210	1.032
Pleural effusion	1.033	0.246	17.612	< 0.001	2.809	1.734–4.550	1.048
CIRP	1.013	0.242	17.538	< 0.001	2.754	1.714–4.424	1.149
CXCL5^a^	0.914	0.271	11.417	0.001	2.495	1.468–4.241	1.196

^a^ Variables showed a nonnormal distribution on the Kolmogorov–Smirnov test and were natural log‐transformed prior to inclusion in the multivariable logistic regression model. For these variables, the OR represents the odds ratio for poor prognosis associated with each 1‐unit increase in the natural logarithm of the variable (corresponding to the original measurement value being multiplied by approximately 2.718). IL‐6 and CIRP were included using their original values; the corresponding OR for each variable represents the odds ratio per 1 pg/mL increase. Categorical variables were coded as follows: MP antibody titer (0 = < 1:160, 1 = ≥ 1:160) and pleural effusion (0, absent; 1, present). The OR for each categorical variable represents the odds ratio relative to the reference group. Extent of pulmonary lesions (1 = single lobe, 2 = two lobes, 3 = > 2 lobes) was treated as an ordinal variable and entered as a continuous variable; the OR represents the odds ratio associated with each additional lobe involved.

### 3.4 Predictive Value of Serum CIRP and CXCL5 for Poor Prognosis in Children With MPP

**FIGURE 2:**
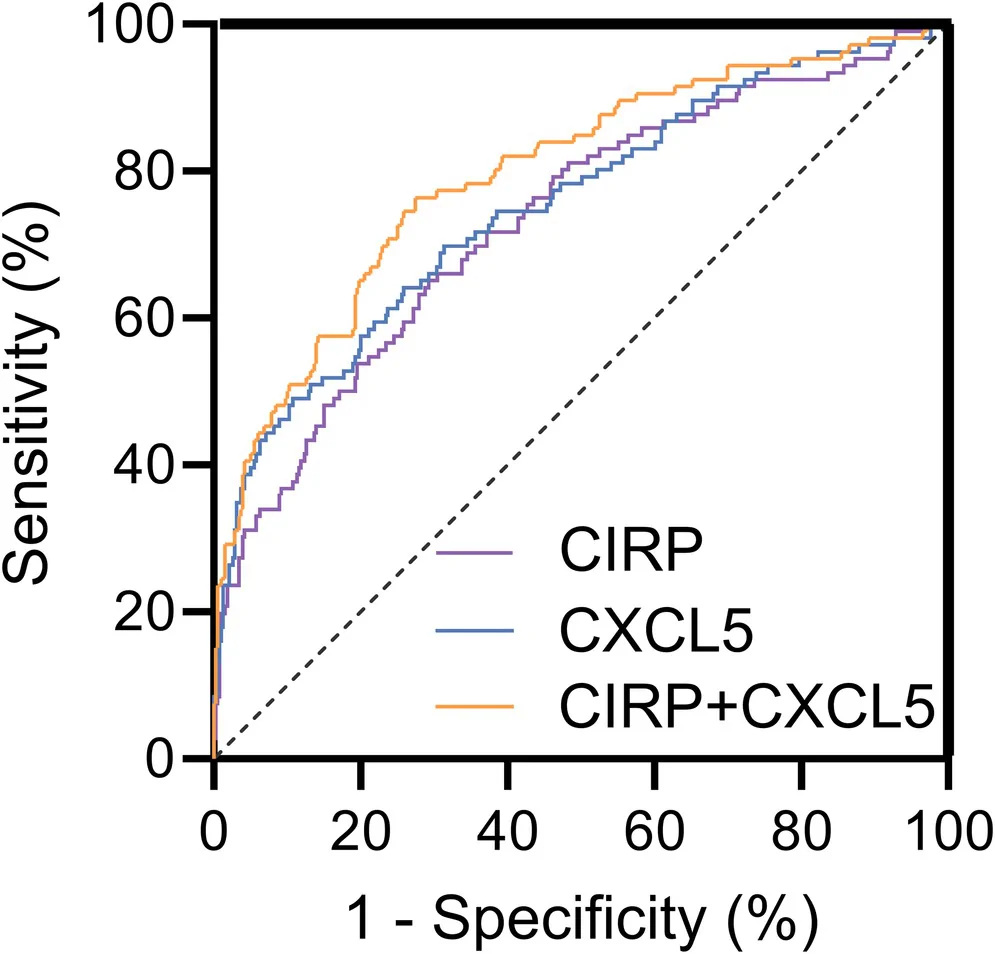
ROC curves of serum CIRP and CXCL5 for predicting poor prognosis in children with MPP.

ROC curve analysis demonstrated that serum CIRP and CXCL5, alone and in combination, had AUCs of 0.733, 0.752, and 0.796, respectively, for predicting poor prognosis in children with MPP (Table 3, Figure 2). The combination exhibited higher sensitivity and specificity than either biomarker alone.

**TABLE 3:** Predictive value of CIRP and CXCL5 for poor prognosis in children with MPP.

Variable	Cutoff value	AUC	95% CI	Sensitivity (%)	Specificity (%)
CIRP	569.60 pg/mL	0.733	0.676–0.789	65.09	70.71
CXCL5	361.40 pg/mL	0.752	0.696–0.808	69.81	68.60
CIRP + CXCL5	—	0.796	0.745–0.847	76.42	72.56

### 3.5 Serum CIRP, CXCL5, and NET Marker Levels and Their Correlations

**FIGURE 3:**
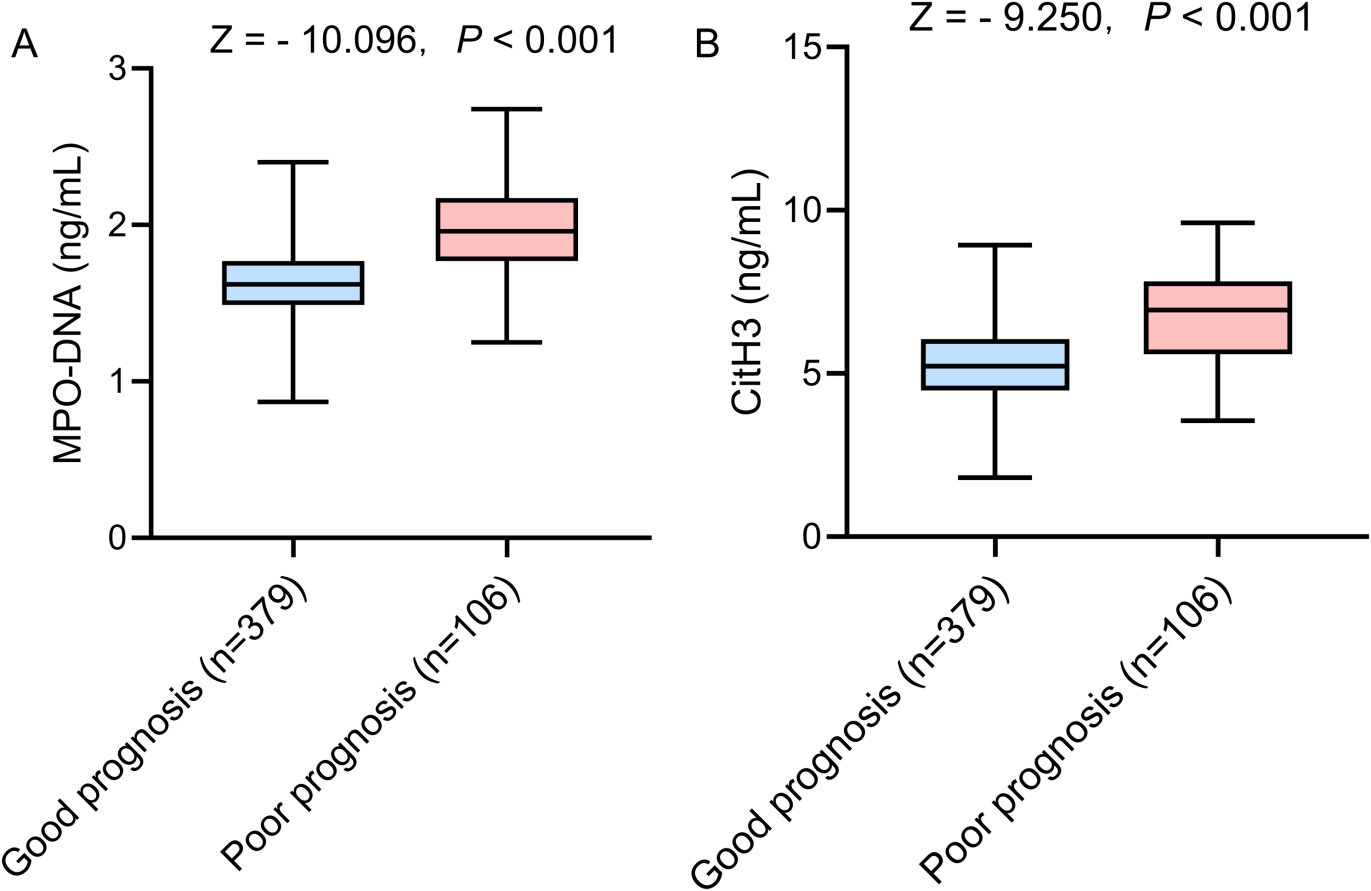
Serum CIRP, CXCL5, and NETs marker levels and their correlations note: (A,B) Comparison of serum MPO‐DNA and CitH3 levels between the poor‐prognosis and good‐prognosis groups; (C,D) correlation of serum CIRP with MPO‐DNA (C) and CitH3 (D); (E,F) correlation of serum CXCL5 with MPO‐DNA (E) and CitH3 (F).

Compared with the good‐prognosis group, serum levels of NET markers (MPO‐DNA and CitH3) were both significantly elevated in the poor‐prognosis group (both *p* < 0.001) (Figure 3A,B). As shown in Table 4, Spearman correlation analysis revealed that in the overall cohort, CIRP and CXCL5 showed moderate positive correlations with NET markers (*r* = 0.411–0.425, all *p* < 0.001); in the good‐prognosis group, weak positive correlations were observed (*r* = 0.301–0.315, all *p* < 0.001); and in the poor‐prognosis group, weak‐to‐moderate positive correlations were found (*r* = 0.340–0.395, all *p* < 0.001). These findings suggest that the concomitant elevation in CIRP, CXCL5, and NET markers is present across all children with MPP but is more pronounced in those with poor prognosis, indicating that this is not a phenomenon specific to the poor‐prognosis subgroup.

**TABLE 4:** Correlations of serum CIRP and CXCL5 with NET markers.

Group	Variable	MPO‐DNA		CitH3	
		r	*p*	r	*p*
Overall cohort (*n* = 485)	CIRP	0.425	< 0.001	0.413	< 0.001
	CXCL5	0.411	< 0.001	0.417	< 0.001
Good‐prognosis group (*n* = 379)	CIRP	0.301	< 0.001	0.302	< 0.001
	CXCL5	0.315	< 0.001	0.313	< 0.001
Poor‐prognosis group (*n* = 106)	CIRP	0.357	< 0.001	0.393	< 0.001
	CXCL5	0.395	< 0.001	0.340	< 0.001

## 4 Discussion

In this study, we measured serum levels of CIRP, CXCL5, and NET‐related markers in children with MPP. We found that serum CIRP and CXCL5 levels were significantly elevated in the poor‐prognosis group, and both markers were positively correlated with levels of NET markers (MPO‐DNA and CitH3). Multivariable logistic regression analysis revealed that CIRP and CXCL5 were independent risk factors for poor prognosis in children with MPP. ROC curve analysis indicated that the combination of these two markers demonstrated favorable predictive value for poor prognosis. These findings suggest that CIRP and CXCL5 may be associated with NET formation and jointly participate in the pathological process of MPP; the combination of these markers may be useful for prognostic evaluation and serve as a potential biomarker for early identification of high‐risk children.

CIRP is a stress‐response protein induced in various cell types under cold stress, hypoxia, and inflammatory stimulation [20]. Under infectious and inflammatory conditions, CIRP can translocate from the nucleus to the cytoplasm and be secreted extracellularly, functioning as damage‐associated molecular patterns (DAMPs) to activate pattern recognition receptors such as Toll‐like receptor 4 (TLR4), thereby initiating downstream NF‐κB and MAPK signaling pathways, promoting the release of pro‐inflammatory cytokines, and amplifying the inflammatory cascade [21, 22]. Previous studies have demonstrated that elevated plasma CIRP levels in patients with severe community‐acquired pneumonia correlate positively with conventional inflammatory markers including PCT, IL‐6, and CRP, as well as the Pneumonia Severity Index score, and effectively predict patient mortality risk [23]. Additionally, CIRP expression is significantly upregulated in normal human bronchial epithelial cells following *S. pneumoniae* infection; mechanistic studies have further demonstrated that CIRP promotes the release of inflammatory factors (such as IL‐6 and TNF‐*α*) via activation of the NF‐κB signaling pathway, thereby contributing to the *S. pneumoniae* ‐induced inflammatory response in bronchial epithelial cells [24]. In the present study, serum CIRP levels were significantly higher in the poor‐prognosis group than in the good‐prognosis group, and CIRP was identified as an independent risk factor for poor prognosis, suggesting that elevated CIRP is closely associated with poor prognosis in children with MPP. The underlying mechanism may be that *M. pneumoniae* infection induces pulmonary and systemic inflammatory responses, leading to tissue damage and substantial release of CIRP into the extracellular space; extracellular CIRP acts as DAMPs to activate the TLR4/NF‐κB and MAPK signaling pathways, further promoting the cascading release of pro‐inflammatory cytokines such as TNF‐*α*, IL‐6, and IL‐1*β*, and exacerbating alveolar‐epithelial apoptosis and interstitial lung injury [25]. Furthermore, in sepsis models, CIRP has been demonstrated to directly induce NET formation through upregulation of PAD4 expression, resulting in acute lung injury; blockade of CIRP or inhibition of PAD4 significantly reduces NET production and improves prognosis [12]. In this study, CIRP was significantly positively correlated with NET markers (MPO‐DNA and CitH3), suggesting a potential association between CIRP and NET formation. Drawing upon mechanisms identified in sepsis models, we speculate that CIRP may participate in the regulation of NET formation through PAD4 activation, thereby contributing to pulmonary inflammatory injury in children with MPP. However, this study employed an observational design, which can only establish correlations among variables rather than prove causality. Whether CIRP directly regulates NET formation, or whether the two merely increase in parallel against a common inflammatory background, remains to be validated in MPP cell models and animal experiments.

CXCL5 belongs to the CXC chemokine family and is a pro‐inflammatory chemokine primarily secreted by epithelial cells, endothelial cells, and macrophages. Through specific binding to its receptor CXCR2, CXCL5 potently chemoattracts neutrophils to inflammatory sites, thereby exacerbating the inflammatory response [14]. Studies have shown that early SARS‐CoV‐2 infection induces massive production of CXCL5 in the lungs, mediating pulmonary inflammatory injury through neutrophil infiltration; conversely, CXCL5 genetic deficiency or antibody neutralization significantly attenuates pulmonary inflammation and pathological damage [16]. In acute lung injury models, CXCL5 knockout mice exhibit significantly reduced neutrophil infiltration, pulmonary edema, and inflammatory cytokine release [15]. Moreover, in a mouse model of chronic obstructive pulmonary disease, treatment with neutrophil elastase inhibitors significantly reduced NET generation in the lungs while simultaneously decreasing CXCL5 levels in bronchoalveolar lavage fluid, suggesting a potential association between NET formation and CXCL5 expression [26]. In this study, serum CXCL5 was significantly elevated in the poor‐prognosis group and positively correlated with NET markers (MPO‐DNA and CitH3), suggesting their joint involvement in the inflammatory process of MPP. The underlying mechanism may be that *M. pneumoniae* infection induces substantial secretion of CXCL5 by alveolar‐epithelial cells and macrophages; neutrophils recruited by CXCL5 are further activated at the site of infection. We hypothesize that CXCL5‐recruited neutrophils may serve as one cellular source of NET formation. However, this hypothesis is based solely on correlative evidence, and the possibility that both may be elevated independently as a common consequence of severe inflammation cannot be excluded. The causal relationship and specific signaling pathways remain to be experimentally verified.

Multivariable logistic regression analysis in this study also identified neutrophil percentage, MP antibody titer, D‐dimer, CRP, IL‐6, extent of pulmonary involvement, and pleural effusion as independent risk factors for poor prognosis in children with MPP, consistent with previous studies [27, 28, 29, 30]. These indicators reflect disease severity across multiple dimensions, including a sustained inflammatory state, infectious burden, coagulation function, and the degree of pulmonary tissue damage; their synergistic effects may accelerate disease progression and increase treatment difficulty. Therefore, for children with MPP presenting with these high‐risk features, early identification and active intervention are warranted, including control of persistent high fever, evaluation of indications for immunomodulatory and anticoagulant therapy, and intensification of respiratory support to further reduce the risk of poor prognosis.

In addition, although traditional inflammatory–coagulation biomarkers such as CRP, D‐dimer, and IL‐6 were identified as independent risk factors for poor prognosis in children with MPP in this study, CRP and IL‐6 represent broad indicators of the systemic acute‐phase response that can be elevated across a wide spectrum of infectious and noninfectious inflammatory conditions and thus lack specificity. D‐dimer primarily reflects activation of the coagulation–fibrinolysis system. In contrast, CIRP and CXCL5 may be more directly linked to neutrophil recruitment and NET release. Therefore, their combination might allow assessment of NET‐related pathological activation; however, this inference is based solely on indirect extrapolation from existing mechanistic studies. Whether this combination truly offers incremental clinical value requires head‐to‐head comparison using the net reclassification index (NRI), integrated discrimination improvement (IDI), and decision curve analysis (DCA), as well as validation in prospective cohorts, to objectively assess its incremental clinical utility.

ROC curve analysis demonstrated that serum CIRP and CXCL5 alone yielded AUCs of 0.733 and 0.752, respectively, for predicting poor prognosis; combined detection of these two markers increased the AUC to 0.796, representing a relatively modest absolute gain. Two potential explanations may account for this observation. First, both CIRP and CXCL5 are molecules involved in neutrophil‐mediated inflammatory pathways and share certain pathophysiological links; consequently, the prognostic information they provide is not entirely independent and partially redundant. Second, in biomarker combination modeling, the incremental improvement in AUC typically follows a pattern of diminishing returns when individual markers already demonstrate moderate discriminatory performance. Nevertheless, compared with either marker alone, the combined assay in this study achieved superior sensitivity (76.42%), specificity (72.56%), and the highest Youden index, suggesting that dual‐marker testing may reduce both false negative and false positive rates in clinical screening. Therefore, combined detection of CIRP and CXCL5 may serve as an adjunctive tool for early identification of high‐risk children with MPP, informing the development of intensified therapeutic regimens or immunomodulatory strategies.

This study has several limitations. First, the single‐center design may introduce selection bias, and multicenter studies with larger sample sizes are needed to validate the generalizability of our findings. Second, macrolide resistance testing was not performed; the poor‐prognosis group may have harbored a higher proportion of resistant strains, and the observed association between elevated CIRP/CXCL5 and poor prognosis may have been partially confounded by macrolide resistance, which could lead to treatment failure. Future prospective studies should incorporate resistance gene testing and perform stratified analyses. Third, given its observational nature, the causal relationships between CIRP, CXCL5, and NETs require further confirmation through cellular experiments and animal models. Fourth, the lack of serial measurements before and after treatment precluded assessment of temporal changes in CIRP and CXCL5 levels during disease progression and determination of whether these markers could serve as indicators for monitoring treatment response. Fifth, the follow‐up duration was relatively short, and long‐term prognostic data were lacking.

In conclusion, this study found that serum CIRP and CXCL5 levels were significantly elevated in children with MPP and poor prognosis; both were independent risk factors for poor prognosis and showed significant positive correlations with levels of NET markers. CIRP and CXCL5 may serve as adjunctive biomarkers for early identification of high‐risk children with MPP; however, the specific mechanisms underlying their association with NETs require further experimental validation, and their clinical translational value remains to be confirmed in prospective studies.

## Author Contributions

**Litao Wan:** conceptualization, data curation, formal analysis, investigation, methodology, software, validation, visualization, writing – original draft, writing – review and editing. **Bolin Liu:** conceptualization, data curation, formal analysis, investigation, methodology, software, validation, visualization, writing – original draft, writing – review and editing. **Ning Xie:** data curation, formal analysis, investigation, methodology, validation, writing – review and editing. **Jianhua Li:** investigation, writing – review and editing. **Ting Su:** investigation, writing – review and editing. **Liyan Zhang:** methodology, resources, writing – review and editing. **Xiaohua Wang:** methodology, resources, writing – review and editing. **Shiding Gao:** conceptualization, methodology, project administration, resources, supervision, writing – review and editing.

## Funding

The authors have nothing to report.

## Ethics Statement

This study involving human participants was approved by the Institutional Review Board of The 908th Hospital of Chinese People's Liberation Army Joint Logistic Support Force (Approval No. 2022‐KY‐07). Written informed consent was obtained from all participants prior to their inclusion in the study. We ensured the confidentiality and anonymity of the participants throughout the research process.

## Conflicts of Interest

The authors declare no conflicts of interest.

## Data Availability

All data in this study are available from the corresponding author on reasonable request.
